# Incidence and Prevalence of Bone Metastases in Different Solid Tumors Determined by Natural Language Processing of CT Reports

**DOI:** 10.3390/cancers17020218

**Published:** 2025-01-11

**Authors:** Niamh Long, David Woodlock, Robert D’Agostino, Gary Nguyen, Natalie Gangai, Varadan Sevilimedu, Richard Kinh Gian Do

**Affiliations:** 1 Department of Radiology, Memorial Sloan Kettering Cancer Center, New York, NY 10065, USA; niamhlong@mater.ie (N.L.); woodlocd@mskcc.org (D.W.);; 2 Department of Radiology, Mater Misericordiae University Hospital, D07 R2WY Dublin, Ireland; 3 Clinical Research Information Technology, Memorial Sloan Kettering Cancer Center, New York, NY 10065, USA; 4 Strategy and Innovation, Memorial Sloan Kettering Cancer Center, New York, NY 10065, USA; 5 Biostatistics Service, Department of Epidemiology and Biostatistics, Memorial Sloan Kettering Cancer Center, New York, NY 10065, USA

**Keywords:** bone metastases, natural language processing, oncology

## Abstract

Background/Objectives: Improved survival due to advances in medical therapy has resulted in increasing numbers of cancer patients living with bone metastases; however, our understanding of the prognostic implications of bone metastases requires larger population-based studies outlining their incidence and prevalence in different primary cancer types, including those with lower incidence. This study aimed to evaluate the incidence and prevalence of bone metastases in solid organ tumors by analyzing reports of staging CT studies with natural language processing (NLP). Methods: In this retrospective study, 639,470 reports representing 129,326 unique patients were analyzed; 6279 randomly selected reports were manually annotated and labeled for the presence or absence of bone metastases. From these data, a BERT-based NLP model was developed and applied to the patient database. The cumulative incidence at 5 years and prevalence of bone metastases in each cancer type were calculated. Results: The accuracy of the NLP model on a validation set was 97.1%, with a positive predictive value (precision) of 88.0% and a sensitivity (recall) of 86.3%. The 5-year incidence rate of bone metastases was highest in prostate, breast, head and neck, and lung cancer (52%, 41%, 36%, 33%). Incidence was lowest in central nervous system cancer and testicular cancer (8%, 5%). Prevalence was highest in prostate, breast, and lung cancer (32%, 25% and 23%), and lowest in central nervous system cancer and testicular cancer (4%, 4%). Conclusions: NLP was utilized to demonstrate patterns of bone metastases in a broad range of cancer types and is a valuable tool in population-based assessment of bone metastases.

## Simple Summary

Bone metastases significantly impact morbidity and mortality in cancer patients, with varying prognostic implications depending on the primary cancer. Understanding patterns of bone metastases across various primary cancer types is vital, yet comprehensive population-based studies are scarce and challenging to execute. Advances in natural language processing (NLP) now make annotations of bone metastasis incidence and prevalence possible on a large scale. We applied NLP to 639,470 CT reports in 129,326 patients, with 97.1% accuracy, 88.0% precision, and 86.3% recall. Primary cancers with the highest incidence of bone metastases on CT reports identified by NLP were prostate and breast cancers, followed by head and neck, and lung cancers, all exceeding a 30% 5-year incidence rate. Other cancers, including melanoma and pancreas cancers, had a 20–30% 5-year incident rate of bone metastases. NLP can be used to accurately label CT reports for the presence or absence of bone metastases on a large scale.

## 1. Introduction

Bone metastases are a common cause of both morbidity and mortality in patients with cancer, at the same time inflicting a great socioeconomic burden globally. In the United States alone, 350,000 people die each year from bone metastases [1]. To date, treatment options for bone metastases have evolved beyond standard surgical care and irradiation. Ablative techniques such as radiofrequency, cryotherapy, and chemoembolization have both tumoricidal and analgesic effects, whereas minimally invasive reconstructive procedures such as cementoplasty and percutaneous screw fixation provide mechanical stabilization. Pharmacological approaches to bone metastases have also evolved beyond treatment with bisphosphonates to monoclonal antibody therapy such as denosumab, with ongoing research into a variety of additional immunotherapeutic options [2].

With the continuing evolution of treatment options for bone metastases, an accurate understanding of disease patterns and their associated implications is vital. While many studies have investigated the incidence of bone metastases in different individual cancers, population-based studies on the incidence and prevalence of metastatic bone disease are lacking. Even among those conducted, the majority have been studies that have derived data from autopsy series which do not reflect current survival trends. A 2020 paper by Huang et al. used the Surveillance, Epidemiology, and End Results (SEER) database to evaluate the incidence and prognosis of bone metastases with solid cancers at the time of cancer diagnosis. This is one of the few population-based studies published on bone metastases in recent years. Indeed, in this paper, the authors state that although approximately 350,000 people die each year in the US from bone metastasis, the incidence rate of bone metastases is still unknown, estimated anywhere between 21,000 and 400,000 per annum [1]. Of note, while the SEER database collects data from multiple cancer registries, it does not directly reflect the pattern of bone metastases identified on computed tomography (CT). Furthermore, the presence of bone metastases has differing prognostic implications in different cancers. Patients with breast and prostate cancer can live for many years with bone metastases; however, in many other cancers, the presence and chronology of bone metastases are powerful prognostic indicators of a shorter survival time [1,3,4]. This is particularly thought to be the case in cancers where bones metastases are less frequently found, such as gastric, hepatobiliary, and anal cancers—all with a median survival time of 3 months following the development of bone metastases [1]. In these cancer types, small numbers of patients with bone metastases limit the ability of traditional study methods to capture significant patient data. Thus, we sought to use novel natural language processing (NLP) techniques to evaluate the incidence and prevalence of bone metastases in multiple solid organ cancer types across a large patient population of more than 120,000 individual patients.

The performance of routine computed tomography (CT) imaging at 2–3-month intervals in patients with cancer who are undergoing either treatment or active surveillance offers radiologists a unique opportunity to harness these data in order to evaluate patterns of skeletal metastatic disease. Radiologists are more likely to identify bone metastases for the first time on CT during staging or follow-up imaging, and their diagnostic interpretation would benefit from CT-based incidence and prevalence data, which are not available from the current literature based on autopsy series or the SEER database. Moreover, while the extraction of data from imaging reports has previously relied on manual methods which are labor-intensive and impractical on a large scale, NLP is increasingly utilized to allow large-scale data extraction and is gaining traction in radiology research [5,6]. There are limited data on the use of NLP to identify bone metastases on imaging reports, including a small study of 704 patients on bone scintigraphy [7], which is used much less often than CT.

Given the relatively scarce data on radiology-reported bone metastases at large scale, the aim of this study was to use NLP to determine the incidence and prevalence of bone metastases across different primary cancers reported at a cancer center using more than a decade of CT radiology reports. This article is a continuation of the authors’ previous study [8] using a larger number of curated reports to improve on a BERT language model. We compared the new model’s performance with preceding approaches and applied the model predictions to a larger number of patients and reports. The novelty of this study lies in the detailed reporting of incidence and prevalence rates of bone metastases across various primary cancer types.

## 2. Materials and Methods

### 2.1. Study Dataset

This retrospective, single-institution study was approved by the institutional review board and a waiver for informed consent was provided. All study activities were conducted in compliance with the United States Health Insurance Portability and Accountability Act.

Consecutive radiology reports for CT examinations of the chest, abdomen, and pelvis, performed between 6 July 2009 and 26 March 2021, were identified from our institutional database via procedure codes. All reports were included if they followed the departmental structured template introduced in July 2009 and contained a “Bones and Soft Tissues” subsection in the “Findings” section, and an “Impression” section. Only primary cancer types with more than 1000 patients were included into the study (20 types of cancers total). An algorithm previously developed at our institution to identify the patients’ most likely primary cancer type at the time of the CT report was applied to the patient database [8]. Reports labeled with any of the following primary cancers were excluded: bone and joint tumors, lymphoma, leukemia, and myeloma. This yielded a total of 639,470 reports which constituted the full study dataset, representing 129,326 unique patients. This study dataset overlapped with 91,655 patients from 2009–2019 that were reported in a different study to demonstrate the feasibility of using NLP to assess spatial and temporal patterns of metastatic spread [8].

For the 639,470 reports, within the “Findings” section of each CT report, the text in the “Bone and Soft Tissues” subsection was extracted along with the text from the “Impression” section. Of note, bone metastases are routinely described by radiologists at our institution in one or both of these sections.

### 2.2. Manual Report Curation

A random sample of 4000 reports from the full study dataset was selected for manual report curation. Manual annotation of these reports was performed by two curators (NL, a radiology attending physician with 7 years of experience; and RD, a research assistant), after both curators completed a training session. In the training session, each curator scored 50 reports, whereby they read the text from both the “Bone and Soft Tissues” subsection and the “Impression” section and then scored the report as either positive or negative for the presence of bone metastases. Both curators were blinded to all other clinical data including imaging while scoring the reports. Specific guidelines were developed for the research assistant to ensure that frequently encountered non-metastatic bone lesions (i.e., degenerative changes, benign bone lesions, or primary bone malignancies) were correctly identified (see the Supplemental Material Table S1 for further details). Following training, the first 1000 reports were scored by both readers, with any discrepancies resolved by consensus. The remaining 3000 reports were divided between the two readers. Manually curated reports that were previously reported [8] and that did not overlap with these new reports were combined for the final dataset of manually annotated reports, yielding a total of 6279 reports.

### 2.3. NLP Model Development

The 6279 manually annotated reports were labeled for the presence or absence of bone metastases and used for NLP model development, as occurred previously [8]. Briefly, the manually annotated dataset was split (80%/20%) into a training and test set to develop the model. The deep learning model was developed in Python 3.7 with the following open-source libraries: NumPy (1.19.1), Pandas (v1.1.1), matplotlib (3.3.1), scikit-learn (v0.23.2), transformers (v3.0.2), PyYAML (v5.3.1), nltk (v 3.5), spaCy (v2.3.2), wordninja (2.0.0), textblob (0.15.3), and PyTorch (v1.6.0). The model was developed in two parts. The first, which accounted for the first 11 neuron layers, was the BERT public language model. The second part was a single classification layer that was trained to suit the underlying classification task. This architecture, which leveraged a pretrained language model, was made available by transfer learning methods and was designed using HuggingFace’s Transformers library. The choice of the BERT language model was based on our initial success with this approach, which had an accuracy of 96.0%, based on training with 2219 annotated reports. The developed model was then applied to the remaining 632,743 reports in the full study dataset, to label for the presence or absence of bone metastases. We also included 448 reports from these remaining reports to form a validation dataset to measure the accuracy of the NLP model. This dataset was generated previously by manual annotation and was randomized to account for demographic variables such as age, race, sex, and proportion of metastatic labels [8]. For frequently occurring short-string texts found in the “Bones and Soft Tissues” subsection, such as “unremarkable”, “no osseous metastases”, or “no osseous lesions”, a rule-based labeling system was also created.

### 2.4. Statistical Analysis

The cumulative incidence of bone metastases at 5 years was estimated using Kaplan–Meier curves, along with their corresponding 95% confidence intervals, which were determined using the log–log approach. The prevalence of bone metastases for each cancer type was determined as the proportion of individuals who were diagnosed with a bone metastasis (based on the NLP model) at any timepoint during the follow-up period. Statistical analyses were performed with SAS 9.4 (SAS Institute Inc., Cary, NC, USA) and R 4.2 (R Core Development Team, Vienna, Austria).

## 3. Results

### 3.1. Study Dataset Characteristics

**Figure 1:**
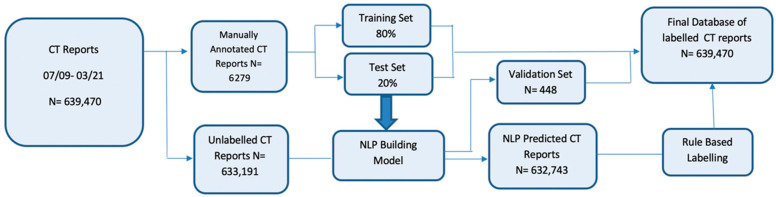
Flowchart detailing the sequential steps in the development of the natural language processing model. There were 639,470 consecutive CT reports first identified between 2009 and 2021. Manual curation was performed on about 1% of reports (6279 report), which were split into training (80%) and testing sets (20%) to build a BERT-based natural language processing model. The final model was evaluated on a validation set of 448 reports and applied to the remaining unlabeled reports. Rule-based labelling was used on a subset of records where the default language (e.g., “unremarkable”) was used in our structured reports.

A total of 639,470 CT reports, performed between 1 July 2009 and 26 March 2021, were included in the dataset, including 73,549/143,218 (51.3%) female patients and 69,655/143,218 (49%) male patients. The mean patient age at the time of the first CT examination was 61.3 (standard deviation, 14.5) years. Each patient had an average of 5 CT studies (range, 1–63). Figure 1 presents a flowchart detailing the sequential steps in the development of the NLP model.

### 3.2. NLP Model Performance

The accuracy of the NLP model on the validation set was 97.1% (435/448), with a positive predictive value (precision) of 88.0% (44/50) and a sensitivity (recall) of 86.3% (44/51).

### 3.3. Incidence Rates of Bone Metastases Across Cancer Types

**Figure 2:**
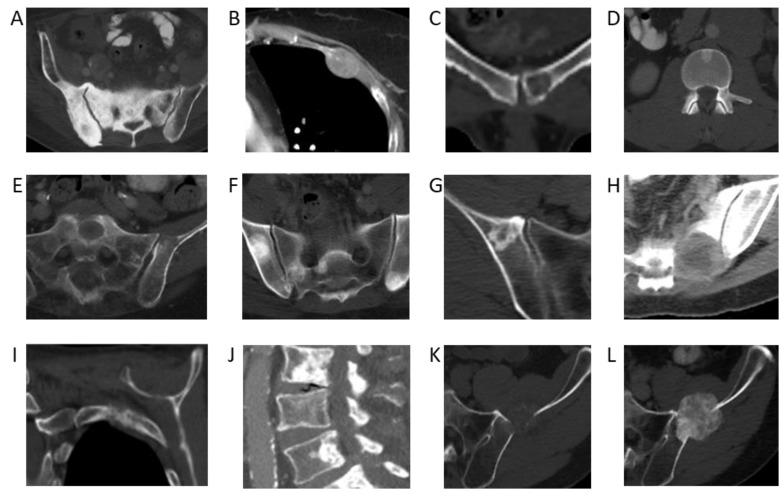
Bone metastases from primary cancers with the highest incidence rates. (**A**) Sclerotic pelvic metastases in a 76-year-old male patient with prostate cancer. (**B**) Lytic metastasis in the left third rib in a 60-year-old female patient with breast cancer. (**C**) Lytic metastasis left pubic bone in a 48-year-old female patient with thyroid cancer. (**D**) Sclerotic metastasis in an L1 vertebral body in a 59-year-old male patient with adenoid cystic carcinoma of the tongue. (**E**) Lytic metastases in the left sacral ala and left ilium in a 65-year-old male patient with poorly differentiated lung adenocarcinoma. (**F**) Multiple sclerotic pelvic bone metastases in a 57-year-old male with a lung carcinoid tumor. (**G**) Sclerotic right iliac metastasis in a 63-year-old female patient with melanoma. (**H**) Lytic left sacral metastasis in a 55-year-old female patient with melanoma. (**I**) Lytic left second rib metastasis in a 60-year-old female patient with hepatocellular carcinoma. (**J**) Multiple lumbar spine sclerotic metastases in a 92-year-old male patient with hepatocellular carcinoma. (**K**) Lytic left iliac metastasis in a 67-year-old male patient with esophageal cancer. (**L**) Follow-up demonstrating interval sclerosis of the left iliac metastasis representing treatment effect.

Using the NLP model, the cancer with the highest 5-year incidence rate of bone metastases was prostate cancer, with an incident rate of 52% (95% confidence interval [CI]: 50–54%). This was followed by breast cancer, with an incident rate of 41% (95% CI: 39–42%), head and neck cancer, with an incidence rate of 36% (95% CI: 32–40%), and lung cancer, with an incidence rate of 33% (95% CI: 32–34%) (Table 1). Incidence rates of bone metastases across melanoma, hepatobiliary, pancreatic, and esophageal cancers were also high (from 20–30%). Examples of CT images from cancers with a high incidence rate of bone metastases are shown in Figure 2.

**Figure 3:**
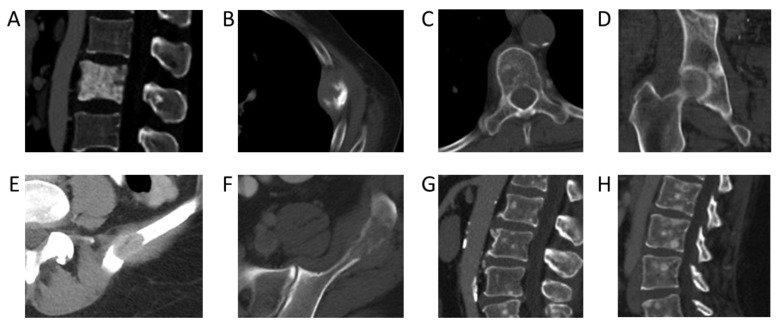
Bone metastases from primary cancers with the highest incidence rates. (**A**) Sclerotic pelvic metastases in a 76-year-old male patient with prostate cancer. (**B**) Left 5th rib lytic metastasis in an 84-year-old male with colorectal cancer. (**C**) Lytic metastasis T5 vertebra in a 64-year-old female patient with ovarian cancer. (**D**) Sclerotic metastasis right acetabulum in a 62-year-old male patient with urothelial cancer. (**E**) Lytic metastasis left ilium in a 54-year-old female patient with renal cell cancer. (**F**) Lytic metastasis left ilium in a 44-year-old male patient with renal cell carcinoma. (**G**) Multiple sclerotic lumbar spine metastases in a 54-year-old male patient with urothelial carcinoma of the renal pelvis. (**H**) Sagittal CT lumbar spine of multifocal sclerotic osseous metastases in a 35-year-old female patient with HER2-negative gastric cancer.

Incidence rates of bone metastases across colorectal, ovarian, genitourinary, and gastric cancers were low (from 10–20%). The lowest incidence rates were found in central nervous system cancer, with an incident rate of 8% (95% CI: 4–11%) and testicular cancer, with an incident rate of 5% (95% CI: 4–6%). Examples of CT images from cancers with a low incidence rate of bone metastases are shown in Figure 3. Prior studies based on autopsy data report higher incidences of bone metastases in breast (67% vs. 41%), prostate (66 vs. 52%), and lung cancer (36 vs. 33%) [8]. This discrepancy can possibly be explained by the fact that patients in our study were evaluated at earlier time points in their cancer journey, whereas autopsy studies are evaluated only once patients have succumbed to their cancer. On the other hand, autopsy studies have reported lower incidences of bone metastases than our NLP-derived results for colorectal cancer (11% vs. 16%) [9,10] and ovarian cancer (9% vs. 11%) [9]. This may be partially attributable to advances in treatment options and associated increased survival, with more patients today living long enough to develop bone metastases. For example, with the introduction of adjuvant treatment regimens, the median survival of patients with advanced colorectal cancer has increased to more than 20 months, with a corresponding increase in the likelihood of developing bone metastases and having adverse skeletal-related events [9,10,11,12,13,14]. The utilization of NLP to extract information from routine CT staging scans affords us the ability to better evaluate the temporal development of bone metastases during a patient’s cancer journey.

### 3.4. Prevalence Rates of Bone Metastases Across Cancer Types

Using the NLP model, the prevalence of bone metastases (the percentage of initial scans with disease) was also highest in prostate cancer, with a prevalence rate of 32%. This was followed by breast cancer, with a prevalence rate of 25% and lung cancer, with a prevalence rate of 23%. Similar to the incidence rates of bone metastases, the lowest prevalence rates were found in central nervous system cancer (4%) and testicular cancer (4%). While incidence rates provide important information regarding overarching patterns of new disease and allow us to evaluate changes over time, prevalence rates are a useful measure of the burden of disease at a specific time point. While many studies in the literature focus solely on the incidence of bone metastases in patients with pancreatic cancer, a SEER study published by Zhang et al. in 2023 evaluated the prevalence and prognosis of bone metastases in common solid cancers at initial diagnosis [15]. Interestingly, this study demonstrated a similar prevalence rate of bone metastases in patients with lung cancer (18.05% vs. 23% in our study) but much lower prevalence rates in patients with breast cancer (3.66% vs. 25%) and patients with prostate cancer (4.61% vs. 32%). These differences are likely explained by the fact that the figures published by Zhang et al. refer to bone metastases at diagnosis, whereas our population was based on those with CTs of the chest, abdomen, and pelvis, which may be obtained at a later stage of treatment.

### 3.5. Bone Metastases-Free Survival Across Cancer Types

**Figure 4:**
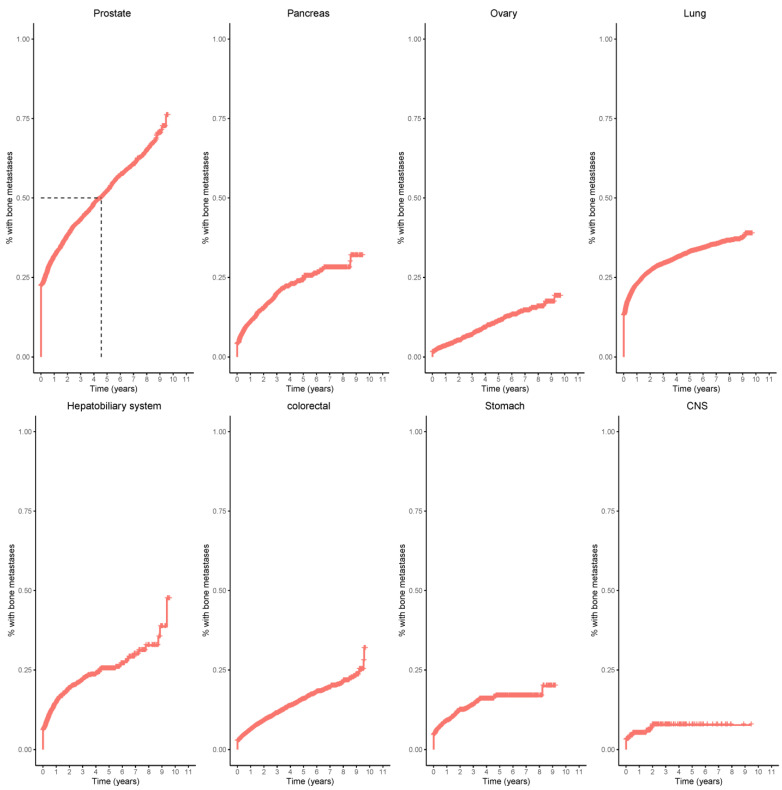
Kaplan–Meier curves of eight primary tumors: prostate, pancreas, ovary, lung, hepatobiliary, colorectal, stomach, and central nervous system (CNS). 50% of patients with prostate cancer developed bone metastases within five years (dashed lines).

Kaplan–Meier curves for the probability of bone metastases illustrate the cumulative incidence rates of bone metastases across various primary cancers (Figure 4).

## 4. Discussion

The temporal patterns of development of bone metastases and their associated impact on patient survival vary greatly depending on the underlying primary cancer type and have also evolved as a result of personalized care. While there are many studies focusing on the presence and implications of bone metastases in cancer types such as breast or prostate cancer, there is a paucity of similar large-scale data on bone metastases in cancer types with a lower incidence of bone metastases, such as colorectal, ovarian, esophageal, pancreatic, and bladder carcinoma [9,16,17,18,19,20,21,22,23,24]. This is due to a variety of factors, including the difficulty in accessing large volume datasets of patients with bone metastases in these cancer types. NLP techniques can supplement traditional data collection methods and are beneficial in data mining of events on a large scale. Thus, we developed an NLP model based on 6279 manually annotated reports for the purpose of labeling reports for the presence or absence of bone metastases on a large scale. The accuracy of the NLP model on the validation set was 97.1%, (435/448) with a positive predictive value (precision) of 88.0% (44/50) and a sensitivity (recall) of 86.3% (44/51). This model outperformed three previously published models [8], the first two based on term frequency–inverse document frequency approaches and a third based on a BERT language model, with accuracy/precision/recall values of Model 1: 92.9%/88.0%/43.1%, Model 2: 94.4%/84.2%/62.8%, and Model 3: 96.0%/88.4%/74.5%. Using the NLP model, the highest incidence and prevalence rates of bone metastases were found in prostate cancer (52%, 32%), followed by breast cancer (41%, 25%). The lowest incidence and prevalence rates were found in central nervous system cancer (8%, 4%) and testicular cancer (5%, 4%).

While we have already highlighted differences in the incidence of bone metastases across various primary tumor types identified by autopsy studies in comparison with our NLP approach, we can also look at how our results compare with larger population-based studies available in the literature. Several population-based studies on the incidence and prevalence of bone metastases have leveraged large patient databases and the SEER database [1,25]. For example, in a review of the SEER database between 2010 and 2016 by Huang et al., which found there were 113,317 cases of bone metastases, among those patients with any metastasis, the rate of bone metastases was 89% in prostate cancer, 54% in breast cancer, and 39% in renal cancer [1]. While rates of bone metastases in that study were evaluated as a percentage of patients with metastatic disease, our study determined incidence rates by also including patients without metastatic disease at the time of baseline imaging. Moreover, databases such as the SEER database are limited by a number of variables, in particular the under-reporting of data and variables in data collection methods. With the widespread use of electronic health records, large volumes of electronic data are readily accessible and can be mined for research purposes.

NLP techniques enable efficient extraction of bone metastases. The use of structured radiology reports further decreases variability in representation of findings, thereby facilitating the adoption and development of NLP models. These techniques are being widely adopted in oncology literature, but to date few studies using NLP techniques have evaluated for the presence of bone metastases. Groot et al. used NLP for binary classification of bone metastases (a single metastasis versus two or more metastases) in 704 bone scintigraphy reports (a much smaller sample size than our own study), attaining a positive predictive value of 97% [7]. Despite the high prevalence of bone metastases in the oncologic population, we believe that our study is the first to evaluate the ability of NLP to calculate the incidence of bone metastases, based on CT imaging, the most common imaging modality used in the follow-up of patients with cancer. The fact that trends regarding incidence of bone metastases calculated by our NLP model are broadly in keeping with previously published data is encouraging and further validates the ability of NLP to process large volumes of data extracted from structured radiology reports.

Whilst it is widely recognized that patterns of bone metastases differ between different cancers, it is increasingly seen that numerous cancers can produce both sclerotic and lytic bone metastases. There is a poor understanding of why patterns of bone metastases can vary widely within specific cancers and across treatment types, both of which we plan to explore within our study database in future studies. We also plan to explore more complex relationships between bone metastases and survival outcomes in different cancer types and the prognostic implications of isolated bone metastases versus bone metastases with co-existing extra-skeletal metastases. Additionally, data regarding the frequency of occurrence of isolated bone metastases in a specific cancer type (i.e., colorectal cancer) would enable radiologists to better characterize incidental bone findings in staging studies. For example, isolated colorectal metastases to the bone are extremely rare; therefore, an isolated bone lesion in a patient with colorectal cancer with no other suspicious findings is highly unlikely to represent a metastasis [8]. Based upon such data, both the avoidance of unnecessary investigations or biopsies and the earlier identification of patients at increased risk of bone metastases and associated skeletal-related events would present obvious benefits in improving patient care and decreasing morbidity. Lastly, for frequently occurring cancer types with a low incidence rate of bone metastases, large-scale NLP studies can enable more accurate evaluation of the prevalence and impact of bone metastases and facilitate the development of guidelines for bone screening and treatment. For example, while ovarian cancer is one of the most common malignancies in women and one of the leading causes of cancer deaths in the United States, the bone accounts for only the fourth common site of metastases behind the liver, lymph nodes, and lung; correspondingly, there are only a few published studies evaluating the incidence and prognostic implications of bone metastases and there are currently no screening guidelines for bone metastases in ovarian cancer [26,27]. Yet, the identification of patients with ovarian cancer who are at higher risk of skeletal-related issues such as pain, cord compression, and pathologic fracture may enable earlier targeted therapies, resulting in improved quality of life for these patients.

Our study had a number of limitations. First, it was limited by its retrospective nature and the sole inclusion of CT imaging. While magnetic resonance imaging (MRI), nuclear bone scan, and more advanced molecular imaging studies such as positron emission tomography/computed tomography (PET/CT) were not labeled in our study, these studies are frequently used for correlation at diagnosis and restaging at our institution. Moreover, not all patients with every type of cancer will undergo CT imaging (e.g., patients with stage 1 tumors or indolent skin carcinoma); therefore, the incidence and prevalence of bone metastases in our study may not be generalizable to all tumor types. However, while the denominators in this study may yield higher percentages of bone metastases than would be found in the entire population of patients with cancer, in a practical sense, few databases are likely to capture all patients with every type of cancer. Second, the single-center design of our study was an additional limitation; however, the adoption of a standardized lexicon by our institution at the time of structured reporting ensured a uniformity of language which decreased the risk of incorrect labeling when employing NLP techniques. Further expansion of our work would require partnering with external centers to investigate the generalizability of our results, using only the “Impression” section of the reports. Third, the use of NLP techniques may change with machine learning methods, but our database is still relatively small for deep learning NLP techniques. Finally, subsequent to the recent launch of ChatGPT, generative artificial intelligence (AI) has attracted a lot of attention with some debate regarding the potential use of such techniques in medical research. Despite the potential advantages of generative AI, there are concerns regarding the potential for inaccurate information, lack of domain expertise in specialized medical fields, and the risk of perpetuating biases present in training data. Additionally, privacy and security concerns related to handling sensitive patient data and challenges in ensuring regulatory compliance are issues that need to be addressed when employing generative AI in medical research. Careful navigation of these issues and limitations is required before integrating generative AI into healthcare research [28].

## 5. Conclusions

In conclusion, in our study, we have demonstrated the value of leveraging NLP techniques to evaluate large numbers of patient data, calculating the incidence and prevalence of metastases to bone with 97% accuracy in more than 120,000 patients with cancer. Our results show a wide range of incidence and prevalence rates of bone metastases across different primary cancers. The 5-year incidence of bone metastases is not surprisingly highest in prostate cancer, at 52%, and less than 10% for primary cancers of the central nervous system and testicular cancer. Prevalence was similarly highest in prostate cancer patients, at 32%, followed by breast cancer and lung cancers, at 25% and 23%, respectively. The single-center, retrospective nature of our study limits the generalizability of our work, but partnership with external institutions to include multi-center data would help overcome this limitation. BERT language models also require large amounts of curated data for training, which was a further limitation. Future work will be centered on the use of large language models as cost, sustainability, and data privacy concerns are addressed in the near future. More study is needed in order to better develop our understanding of patients at the greatest risk of bone metastases and of the prognostic implications of bone metastases across different cancer types. This study will provide the basis upon which we plan to evaluate these relationships, which will better inform clinicians directing both diagnostic imaging protocols and targeted therapeutic interventions.

## Figures and Tables

**Table 1:** Five-year incidence rates for 18 of the most common cancer types.

Tumor Type	5-Year Incidence Rate	95% Confidence Interval
Prostate	52%	50–54%
Breast	41%	39–42%
Head and Neck	36%	32–40%
Lung/Bronchus	33%	32–34%
Melanoma	27%	25–29%
Hepatobiliary	25%	23–28%
Thyroid/Endocrine	25%	22–27%
Genitourinary (Renal & Bladder)	24%	23–25%
Pancreas	24%	22–27%
Esophagus	23%	21–26%
Cervix	17%	13–21%
Gastric	17%	14–20%
Uterus	16%	14–18%
Colorectal	16%	15–17%
Ovary	11%	10–13%
Small Bowel	10%	8–13%
Central Nervous System	8%	4–11%
Testicular	5%	4–6%

## Data Availability

Data generated or analyzed during the study are available from the corresponding author by request.

## Supplementary Materials

The following supporting information can be downloaded at https://www.mdpi.com/article/10.3390/cancers17020218/s1, Table S1: Wording unrelated to metastatic disease.

## Abbreviations

AI, artificial intelligence; CNS, central nervous system; CT, computed tomography; MRI, magnetic resonance imaging; PET/CT, positron emission tomography/computed tomography; SEER, Surveillance, Epidemiology, and End Results.
