# Supplementary material for: Incidence and Prevalence of Bone Metastases in Different Solid Tumors Determined by Natural Language Processing of CT Reports
Source: Cancers (Basel). 2025 Jan 11;17(2):218. doi: 10.3390/cancers17020218 (PMC11763382; doi:10.3390/cancers17020218)
Supplement: Supplementary file 1 [file cancers-17-00218-s001.zip › cancers-3313048-supplementary.pdf]

Supplementary Material

Table S1. Wording unrelated to metastatic disease. To identify osseous metastatic disease within the bones/soft tissues section text: 1) ignore findings related to soft tissues (e.g., muscles, cutaneous tissues); 2) ignore wording unrelated to metastatic disease, for example, sentences related to degenerative disease, describing benign bone findings, and primary bone malignancies.

Related to degenerative disease	Benign bone findings	Primary bone malignancies
Avascular necrosis	Aneurysmal bone cyst	Chondrosarcoma
Bone infarct	Bone Island	Chordoma
Dextrocurvature, levocurvature, scoliosis	Chondroblastoma, chondroid lesion, enchondroma	Ewing's sarcoma
Degenerative disc disease, degenerative joint disease	Eosinophilic granuloma	Fibrosarcoma
Endplate sclerosis	Fibrous cortical defect, fibrous dysplasia	Giant cell tumor
Facet joint hypertrophy	Geode	Multiple myeloma
Kyphosis/lordosis	Giant cell tumor	Osteosarcoma
Osteoarthrosis, osteoarthritis	Hemangioma	Primary bone lymphoma
Osteophytes, enthesophytes	Intraosseous lipoma	
Pars defect	Liposclerosing myxofibrous tumor (LMSFT)	
Schmorl's node	Non-ossifying fibroma	
Spondylolysis	Osteoid osteoma, osteoblastoma	
Spondylolisthesis	Solitary bone cyst	
Subchondral cyst, subchondral cystic changes, subchondral sclerosis	Punctate or subcentimeter sclerotic lesion	
